# Review of light field technologies

**DOI:** 10.1186/s42492-021-00096-8

**Published:** 2021-12-03

**Authors:** Shuyao Zhou, Tianqian Zhu, Kanle Shi, Yazi Li, Wen Zheng, Junhai Yong

**Affiliations:** 1 Y-tech, Kuaishou Technology, Beijing, 100085 China; 2 EECS Department, University of California, Berkeley, CA 94720 USA; 3 School of Software, BNRist, Tsinghua University, Beijing, China

**Keywords:** Light field imaging, Holographics, Human-machine graphic interaction

## Abstract

Light fields are vector functions that map the geometry of light rays to the corresponding plenoptic attributes. They describe the holographic information of scenes by representing the amount of light flowing in every direction through every point in space. The physical concept of light fields was first proposed in 1936, and light fields are becoming increasingly important in the field of computer graphics, especially with the fast growth of computing capacity as well as network bandwidth. In this article, light field imaging is reviewed from the following aspects with an emphasis on the achievements of the past five years: (1) depth estimation, (2) content editing, (3) image quality, (4) scene reconstruction and view synthesis, and (5) industrial products because the technologies of lights fields also intersect with industrial applications. State-of-the-art research has focused on light field acquisition, manipulation, and display. In addition, the research has extended from the laboratory to industry. According to these achievements and challenges, in the near future, the applications of light fields could offer more portability, accessibility, compatibility, and ability to visualize the world.

## Introduction

**Fig. 1:**
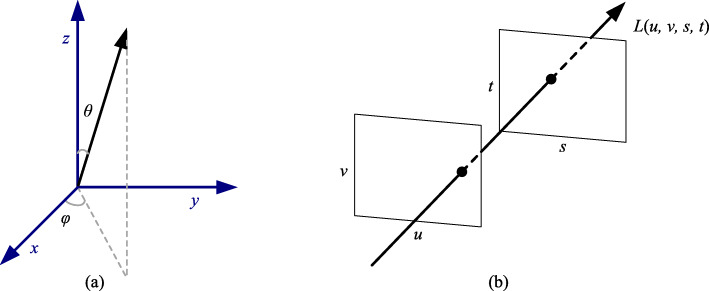
5D and 4D light field representations. **a** *L*(*x*, *y*, *z*, *θ*, *φ*) ∈ *R*^5^, where (*x, y, z*) represents the coordinates, and (*θ*, *φ*) represents the angles between the light ray and the planes; **b** *L*(*u*, *v*, *s*, *t*) ∈ *R*^4^, where (*u, v*) represents the first plane and (*s, t*) the second plane

**Fig. 2:**
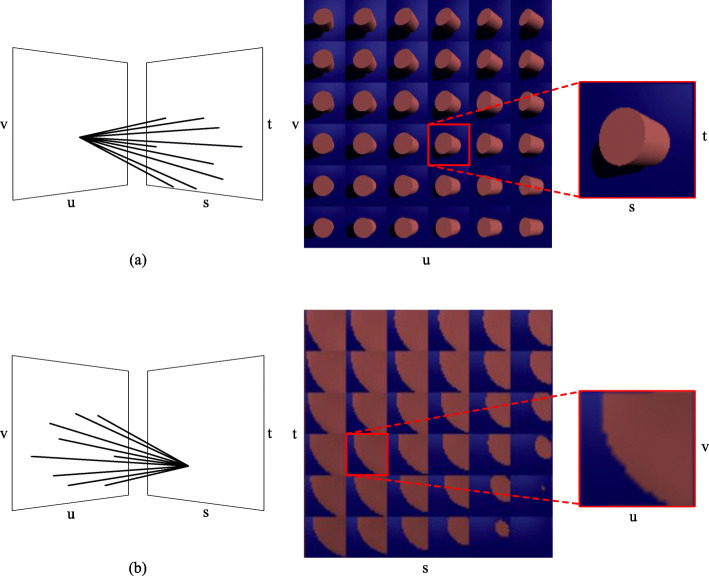
Two visualizations of a light field. **a** Each image represents all the light rays leaving the *st* plane that can pass the same point on the *uv* plane; **b** Each image represents the light rays leaving from the same point on the *st* plane to different points on the *uv* plane

A light field is the totality of light rays or radiance in three-dimensional (3D) space through any position and in any direction, as defined by Gershun [1] in 1936. Formally,

$$ L:g\to c $$

where a light field *L* is defined by mapping the geometry of a light ray *g* to the attributes of the corresponding light *c*. Here, *c* is a vector that describes the intensity of every component of the light such as red, green, and blue (RGB). Geometrically, *g* has various definitions in different light field models. A plenoptic function describes all visual information [2]. Gershun [1] defined a five-dimensional (5D) plenoptic function *L*(*x*, *y*, *z*, *θ*, *φ*) ∈ *R*^5^ for the light field because each ray can be parameterized by three coordinates (*x*, *y*, *z*) and two angles (*θ*, *φ*). Compared with the previous 5D representation, Levoy and Hanrahan [3] assumed in their four-dimensional (4D) representation *L*(*u*, *v*, *s*, *t*) ∈ *R*^4^ that the light field is composed of oriented lines in free space, successfully reducing the redundancy of the total dataset and simplifying the reconstruction of the plenoptic function. *L*(*u*, *v*, *s*, *t*) parameterizes lines by their intersections with two planes in an arbitrary position, where (*u*, *v*) represents the first plane and (*s*, *t*) represents the second plane (see Fig. 1 for the 5D and 4D light field representations and Fig. 2 for two different visualizations of the light field). Meanwhile, Levoy and Hanrahan introduced light fields to the computer graphics field. In addition, if one describes a light field captured by a camera moving on a sphere centered on the target object, then geometry g can be defined as (*θ*, *φ*, *s*, *t*) ∈ *R*^4^, where (*θ*, *φ*) ∈ *R*^2^ is a spherical surface and (*s*, *t*) ∈ *R*^2^ is a plane surface with light projecting to it. “Ray space” is a synonym of “light field” [4, 5] to describe rays in a 3D space. The light field is the same as the orthogonal ray space. In the field of free-viewpoint televisions [6, 7], the term “ray space” is often used to describe ray-based 3D information display systems.

**Fig. 3:**
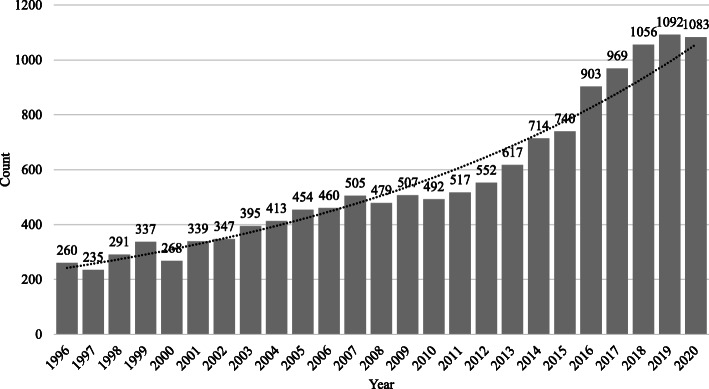
Light field imaging timeline: numbers of publications whose titles contain keywords “light field” or “light fields” or “light-field” or “ray space” from 1996 to 2020 (the dotted line is the geometric approximation)

In 2005, the light field made the transition from mainly pure research to large-scale industrial applications. For example, Ng et al. [8] developed the first handheld plenoptic camera. It was not until 2010 that light field technology was commercialized to capture a light field. With the development of commercial light field cameras [9], plenoptic cameras that make it possible to refocus provide many benefits, and they have been widely used in light field applications. Subsequently, the commercial potential of the light field has been greatly illustrated in image editing, holographically perceived light fields, augmented reality, and physical entities such as plenoptic cameras. The number of publications has increased geometrically as light fields have gained increasing attention from researchers (see Fig. 3 for the timeline of light field imaging).

Light field acquisition is the preliminary light field imaging process. Wu et al. [10] comprehensively highlighted methods and devices for light field acquisition in their survey, including (1) multisensor capture (using multiple cameras to capture a light field at one time, with most of them being camera arrays [11–17]), (2) time-sequential capture (using one camera to capture a light field with multiple exposures, which is time consuming [18–23]), and (3) multiplexed imaging [encoding high-dimensional data into a simpler two-dimensional (2D) image, which is the most popular method [8, 24–37].

**Fig. 4:**
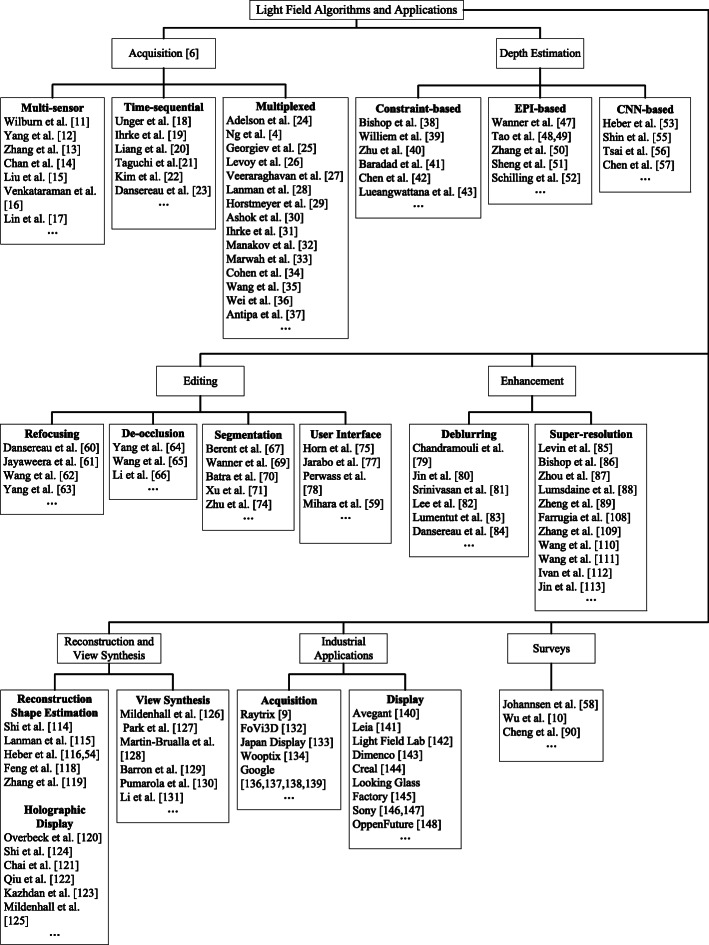
Overview of light field algorithms and applications: the organization graph shows acquisition methods (Introduction Section), current depth estimation methods (Depth estimation Section), light field editing techniques (Editing Section), light field enhancements with an emphasis on increasing the quality of images (Enhancement Section), 3D reconstruction and view synthesis (Reconstruction and view synthesis Section), light field industry, which is categorized into light field acquisition and light field display (Industrial applications Section), and past surveys on light fields

Herein, this paper aims to review light field imaging, revealing the current deficiencies and exploring the future possibilities (see Fig. 4 for an overview). Five aspects have been reviewed: current depth estimation methods (Depth estimation Section), light field editing techniques (Editing Section), light field enhancements with an emphasis on increasing the quality of images (Enhancement Section), 3D reconstruction and view synthesis (Reconstruction and view synthesis Section), and the light field industry, which is categorized into light field acquisition and light field displays (Section 6).

### Depth estimation

Depth estimation involves inferring 3D information from 2D images, which is a foundation for light field editing and rendering. Light field data record the spatio-angular information of light rays; thus, a light field image contains many depth cues to make depth estimation possible. Conventionally, depth cues include, but are not limited to, correspondence cues, defocus cues, binocular disparity, aerial perspective, and motion parallax. Occlusion often occurs when two or more objects come too close and, therefore, hide some information from each other. Specifically, when people want to see an occluded object, they usually move slightly to avoid the occluder. This commonsensical solution explains why light fields have special benefits in solving the depth map with occlusion. Therefore, researchers have mainly focused on traditional approaches and convolutional neural network (CNN) approaches for depth estimation with examinations of occlusion handling: (1) constraint-based estimation, (2) epipolar plane image (EPI)-based estimation, and (3) CNN-based estimation.

Constraint-based estimation utilizes different constraints of the light field structure to estimate the depth. Bishop and Favaro [38] estimated the depth from multiple aliased views and demonstrated that this could be done at each pixel of a single light field image. Williem and Lee [39] utilized the correspondence cue and defocus cue, which were robust against both occlusion and noise, and they introduced two data costs: the constrained angular entropy cost and constrained adaptive defocus cost. Zhu et al. [40] addressed a multioccluder occlusion by regularizing the depth map with an antiocclusion energy function. Some researchers have considered the relationship between occluders and natural light reflections. For example, Baradad et al. [41] estimated the 4D light field of a hidden scene from 2D shadows cast by a known occluder on a diffuse wall by determining how light, which naturally reflected off surfaces in the hidden scene, interacted with the occluder. Chen et al. [42] detected partially occluded boundary regions (POBRs) by using superpixel-based regularization. After a series of shrinkage and reinforcement operations on the labeled confidence map and edge strength weights over the POBR, they produced a depth estimate with a low average disparity error rate and high occlusion boundary precision-recall rate. To proceed with occlusion handling from image to video, Lueangwattana et al. [43] examined the structure from motion to improve light field rendering and, hence, addressed fence occlusion in videos while preserving background details.

However, the EPI, proposed by Bolles et al. [44] in 1987, simplifies depth measurement by restricting motion to straight lines and working with a series of closely spaced images, thereby reducing the 3D problem into a set of 2D problems. Some studies on EPI representations are highlighted here. Matoušek et al. [45] suggested a dynamic-programming-based algorithm find correspondences in EPIs by extracting lines with similar intensities in an EPI separately for each row. In other research, Criminisi et al. [46] worked with EPI volume, a dense horizontally rectified spatio-temporal volume that results from a linearly translating camera, for automated layer extraction. They relied on an EPI tube, which is a collection of EPI lines of the same depth.

Unlike the above works that refine EPI representations, the following works established how to apply EPIs in depth estimation. Wanner and Goldluecke [47] used the dominant directions of EPIs from the structure tensor method to estimate depth. However, this method is sensitive to noise and occlusion. In addition, estimation based on a 2D EPI is vulnerable to noise and sometimes fails because of very dark and bright image features.

Multiorientation EPIs are epipolar plane images in all available directions and provide rich light field angular information. To achieve a better depth map from light field images, Tao et al. [48] computed dense depth estimation by combining defocus and correspondence depth cues based on full 4D EPI. Moreover, defocus cues perform better in repeating textures and noise, and correspondence cues are robust in terms of bright points and features. Tao et al. [49] obtained defocus cues by computing the spatial variance after angular integration and correspondence depth cues by computing the angular variance. Furthermore, they performed depth estimation on glossy objects with both diffuse and specular reflections and one or more light sources by exploiting the full EPIs. Similarly, Zhang et al. [50] proposed a spinning parallelogram operator (SPO) to locate lines and calculate their orientations in an EPI for local depth estimation, which further handled occlusions and was more robust to noise. In addition, Sheng et al. [51] combined a multiorientation SPO with edge orientation to improve depth estimation around occlusion boundaries. They proved that the direction of the optimal EPI was parallel to the boundary of the occlusion. In contrast to the work of Sheng et al. [51], Schilling et al. [52] incorporated both depth and occlusion using an inline occlusion-handling scheme, OBER-cross+ANP, to improve object boundaries and smooth surface reconstruction.

Deep CNNs have been extensively applied to depth estimation because they have a better balance between accuracy and computational cost. In 2017, Heber et al. [53] extended the previous work [54] in which the network operated on EPIs, and they replaced all 2D operators with 3D counterparts. Then, they used a CNN that predicted disparity based on RGB EPI volumes, and the proposed network learned to recover depth information for shape estimation. For supervised training, researchers require large labeled datasets. Shin et al. [55] solved the data insufficiency problem by incorporating a multistream network, which encoded each EPI separately for depth estimation, into their CNN model EPINET. Tsai et al. [56] proposed an attention-based view selection network that exploited the priorities of light field images and the correlations between them to reduce redundancy and computation time. In 2021, Chen et al. [57] applied an attention-based multilevel fusion network. They grouped four directions (0°, 45°, 90°, and 135°) of light fields into four branches. Then, they combined the branches with two feature fusion methods to generate depth maps: intrabranch feature fusion based on channel attention and interbranch feature fusion based on branch attention. Researchers usually employ deep CNNs for accurate depth estimation, combining them with traditional approaches to produce better results.

Depth estimation has been a focus of much research. Researchers have worked on constraint-based methods and have explored different depth cues and their combinations. They also simplified the estimation by using EPIs and applying learning-based methods. There have been other studies that evaluate depth estimation methods. The work of Johannsen et al. [58] covers more depth estimation algorithms before and including 2017. The key to enhancing other light field-related applications, such as refocusing or rendering, is to develop more-precise and more robust depth estimation methods.

### Editing

Because most light field datasets contain redundancy, researchers are interested in fully using redundancy and manipulating the light field images. Editing light fields is challenging [59] because (1) the light fields are 4D, whereas most tools on the market are for 2D, (2) local edits need to preserve the redundancy of the 4D light field, and (3) the depth information of the 4D light field is implicit. Light field image editing can be divided into (1) refocusing, (2) removing the occlusion, (3) segmenting the light fields to make the editing experience as smooth as editing a 2D image (e.g., removing the scene objects or changing their color), and (4) improving the user interface of the light field editing.

Because light field images contain not only textural information but also geometrical information, researchers can explore refocusing after capturing that cannot be accomplished with 2D images. In 2015, Dansereau et al. [60] demonstrated that a hyperfan-shaped passband can achieve refocusing over a wide range of depths, which they called “volumetric refocusing.” However, the approach only worked for a single volumetric region. To overcome this, Jayaweera et al. [61] proposed a simultaneous refocusing approach for multiple volumetric regions in light fields. They employed a 4D sparse finite-extent impulse response filter, which is a series of two 2D filters composed of multiple hyperfan-shaped passbands. Noncrucial parts of images produced by digital single-lens camera arrays often experience blurs (bokeh). Wang et al. [62] proposed a light field refocusing method to improve bokeh rendering and image quality. They first estimated the disparity map and rendered the bokeh on the center-view sub-image. The rendered bokeh image was then used as a regularization term to generate refocused images. Moreover, Yang et al. [63] proposed a refocusing framework that produced coordinates for interpolation, and they aligned the images onto the focal plane.

Occlusion removal is another typical task in light field editing. The nature of light field sub-aperture images (SAIs) provides complementary information so that hidden scenes can be seen from other views. Yang et al. [64] partitioned an image into multiple visibility layers and propagated the visibility information through layers. The visibility layer is defined as all the occlusion-free rays in any camera, computed by energy minimization. For CNN methods, Wang et al. [65] suggested a deep encoder-decoder network for automatically extracting foreground occlusions by analyzing scene structures. The SAIs were first encoded with spatial and angular information and then decoded for center-view reconstruction. However, they only considered the one-dimensional (1D) connections among the SAIs. To improve this, Li et al. [66] proposed another CNN-based encoder–decoder method (Mask4D) to learn the occlusion mask with center-view reconstruction. They applied a 5D tensor to explore spatial connections among SAIs. Occlusion removal is also useful for reconstruction.

Segmentation is a specific research focus in light field editing. Berent and Dragotti [67] proposed an algorithm to extract coherent regions based on a level set method [68]. Wanner et al. [69] carried out globally consistent multilabel assignment for light field segmentation. It used appearance and disparity cues, similar to the multiple-view object segmentation method developed by Batra et al. [70], a method that could automatically segment calibrated images from multiple viewpoints with an energy minimization framework that combined stereo and appearance cues. Xu et al. [71] proposed an approach for localizing transparent objects in a light field image. They used light field linearity, the linearity of the light field distortion feature, which modeled refraction in objects between views captured by a light field camera, to separate Lambertian objects (good light field linearity) and transparent objects (poor light field linearity), and they found the occlusion area by using the occlusion detector, which detected occlusion points by checking the consistency of the forward and backward matches between a pair of viewpoints. As a result, the method could finish the transparent object segmentation automatically without any human interaction.

Superpixel algorithms [72] group pixels into perceptually meaningful atomic regions, which can be used to replace the rigid structure of the pixel grid. Previous methods of segmenting 2D images, such as the simple linear iterative clustering superpixels [73], adopted k-means for superpixel generation. As mentioned in the first paragraph of this section, there are three difficulties with light field editing. In 2017, Zhu et al. [74] defined a light field superpixel as a light ray set that contains all rays emitted from a proximate, similar, and continuous surface in the 3D space, and they essentially eliminated the defocus and occlusion ambiguities in traditional 2D superpixels. Unlike in previous works, they focused on a smaller unit — the superpixel — illustrating that superpixel segmentation on a 4D light field performed better in representing the proximity regions.

For existing user interfaces, users can employ tools to edit the light field, even though the depth map is imperfect. Horn and Chen [75] designed LightShop to manipulate light fields by operating on view rays. Image warping is a process of distorting an image [76]. When a user defines how the view rays warp, the LightShop renderer composites and renders multiple light fields by executing the user-defined ray-shading program. However, the system has limitations when compositing different light fields because of the fixed illumination of a light field. In 2014, Jarabo et al. [77] provided an overview of different light field editing interfaces, tools, and workflows from a user perspective. Some products, such as the single-lens 3D-camera with extended depth of field presented by Perwaß and Wietzke [78], can refocus or apply predefined filters to light field images. Building on previous work [75], Mihara et al. [59] were the first to use a graph-cut approach for 4D light field segmentation based on a learning-based multilabel segmentation scheme. The user needs to specify a target region so that the algorithm can identify the appropriate regions and evaluate whether each ray is included in the selected region. Moreover, they defined appropriate neighboring relationships to preserve redundancies.

Overall, light field editing methods remain largely unexplored compared with 2D image editing. Researchers have experimented further on light field images, such as adding mosaic or special effects or filters, editing body features, combining several images into a short movie, and changing background, which are promising.

### Enhancement

Light field enhancement optimizes the quality of light field images. Modern light field research mainly focuses on deblurring and super-resolution (SR).

Motion deblurring has two approaches: blind motion deblurring and nonblind deblurring. Blind motion deblurring has been extensively studied using 2D images. In 2014, Chandramouli et al. [79] first investigated motion deblurring for light field images and assumed constant depth and uniform motion for the simplicity of the model. Jin et al. [80] explored bilayer blind deconvolution that removed the motion blur in each layer to recover 2D textures from a motion-blurred light field image. Moreover, Srinivasan et al. [81] introduced a light field deburring algorithm by analyzing the motion-blurred light field in the primal and Fourier domains. Unlike in previous studies, they recovered a full 4D light field image. Lee et al. [82] addressed six-degree-of-freedom (6-DOF) blind deblurring by considering the 3D orientation change of the camera. Lumentut et al. [83] proposed a deblurring deep neural network with 16,000 times faster speed than in prior work [81] and deblurred a full-resolution light field in less than 2 s. Dansereau et al. [84], unlike in the above approaches, adopted a nonblind algorithm for 6-DOF motion blur, assuming that the ground truth camera motion was known.

Levin et al. [85] illustrated that there is a trade-off between spatial and angular resolutions. Therefore, many researchers have endeavored to improve the spatial resolution of the captured light field using an SR algorithm by exploiting additional information from the available data. For instance, in 2009, Bishop et al. [86] studied SR algorithms using a depth map. They characterized the point-spread function of a plenoptic camera under Gaussian optics assumptions for a depth-varying scene and formulated the reconstruction of the light field in a Bayesian framework. Therefore, they restored the images at a resolution higher than the number of microlenses. Zhou et al. [87] applied the ray-tracing method to analyze the subpixel shifts between the angular images extracted from the defocused light field data and the blur in the angular images, and they obtained an SR result with a magnification ratio of 8. In contrast to restoring high-resolution images from low-resolution images, Lumsdaine and Georgiev et al. [88] rendered high-resolution images by adopting positional and angular information in captured radiance data. They rendered images from a 542-megapixel light field to produce a 106-megapixel final image. Zheng et al. [89] presented a convolutional deep neural network using cross-scale warping to the reference-based SR, which involves applying an extra high-resolution image as a reference to help super-resolve a low-resolution image that shares a similar viewpoint. In 2019, Cheng et al. [90] categorized the existing SR methods into projection-based [91–94], optimization-based [95–101], and learning-based [102–107]. Moreover, Farrugia and Guillemot [108] reduced the light field angular dimension using low-rank approximation and then applied CNNs to achieve peak signal-to-noise ratio (PSNR) gains of 0.23 dB over the second-best-performing method. Zhang et al. [109] proposed a residual convolutional network for higher spatial resolution. They first improved the spatial resolution of the central view image because it contained more subpixel information. Then, they trained the network to improve the spatial resolution of the entire light field image. Wang et al. [110] suggested a spatial-angular interactive network. They began by extracting the spatial and angular features independently from the input light field images, and then the information was processed by many interaction groups to achieve spatial-angular interaction features. Finally, the interacted features were fused to achieve high-resolution SAIs. Similar to previous researchers [110], who used feature collection and distribution, Wang et al. [111] proposed a deformable convolution network where all side-view features were aligned with the center-view feature and then aligned with the original features. Consequently, angular information is encoded into views for SR performance. Ivan and Williem [112] investigated an end-to-end encoder-decoder style for a joint spatial and angular light field SR model from only a single image without relying on physical-based rendering or secondary networks so that end users can experience the advantages of light field imaging. Jin et al. [113] proposed another learning-based light field spatial SR framework that uses deep combinatorial geometry embedding and structural consistency regularization. Their method improved the average PSNR by more than 1.0 dB and preserved more-accurate parallax details at a lower computational cost.

Researchers have devoted great effort to light field image deblurring and resolution trade-offs. For individual users, the capturing process involves more randomness, which requires stabilization, and they expect to achieve a high-resolution display similar to what they can obtain from a normal camera.

### Reconstruction and view synthesis

Geometric reconstruction involves reconstructing an object from its geometric information. Sparsity in the Fourier domain is an important property that makes 4D light field reconstruction possible from a small set of samples. Shi et al. [114] proposed a method for recovering non-Lambertian light fields from a small number of 1D viewpoint trajectories optimized for sparsity in the continuous Fourier domain. In general, 3D reconstruction includes shape estimation, and some research has extended the results to holography. In addition, view synthesis creates new views from a given set of views.

For shape estimation, Lanman et al. [115] proposed surround structured lighting to achieve full 360° reconstructions using a single camera position, but it could only scan relatively small volumes. Subsequently, multiview reconstruction methods have become increasingly popular. Heber et al. [116] proposed a variational multiview stereo method, in which they used a circular sampling scheme inspired by a technique called “active wavefront sampling” (AWS) [117], where the AWS module is an off-axis aperture that moves along a circular path around the optical axis. In 2016, Heber and Pock [54] trained a CNN to predict 3D scene points from the corresponding 2D hyperplane orientation in the light field domain by using horizontal and vertical EPIs and dividing each EPI into patches. Similarly, Feng et al. [118] focused on 3D face reconstruction from 4D light field images using a CNN. They constructed 3D facial curves, rather than a complete face, to make up a 3D face at once by combining all the horizontal and vertical curves of a face to form horizontal and vertical depth maps separately. However, unlike Heber and Pock [54], they exploited a complete EPI for depth prediction. Zhang et al. [119] applied a light field camera as a virtual 3D scanner to scan and reconstruct 3D objects, which enabled dense surfaces to be reconstructed in real time. They illustrated that, with five light fields, the reconstructed 3D models were satisfactory.

Another application of light field 3D reconstruction is the holographically perceived light field because the light field can provide continuous focus cues. Overbeck et al. [120] developed “welcome to light fields” to enhance the virtual reality experience by setting up a 16-GoPro rotating rig, processing multiview depth maps, adopting disk-based light field rendering to make seamless connections among pictures, and varying compression levels with the movement of the eyeballs. Many studies have demonstrated high-quality scene rendering [121–123]. In 2017, Shi et al. [124] introduced near-eye light field computer-generated rendering with spherical waves for wide-field-of-view interactive 3D computer graphics. Nonetheless, Mildenhall et al. [125] proposed a deep-learning method for view synthesis from an irregular grid of sampled views that first expands each sampled view into a local light field by means of a multiplane image scene representation and then blends adjacent local light fields. They used up to 4000 times fewer views and presented a plenoptic sampling framework by clearly specifying how users should sample input images for view synthesis with portable devices.

Recent view synthesis methods have approached new view generation from only a few input images. The neural radiance field (NeRF) [126] is a nonconvolutional deep network representation that characterizes the volume space with a multilayer perceptron. It takes a continuous function (*x*, *y*, *z*, *θ*, *φ*) ∈ *R*^5^ as input, and it outputs the volume density and view-dependent RGB color. Overall, it reconstructs the surface geometry and appearance from a small set of images. Subsequent studies have extended the NeRF in a variety of ways. Park et al. [127] modeled shape deformations by augmenting the NeRF. They can reconstruct free-viewpoint selfies from photographs. Nonetheless, the NeRF in the wild [128] considers the photometric and environmental variations between images to reconstruct real-world scenes. Compared with NeRF, Mip-NeRF [129] takes in a 3D Gaussian that represents the region over which the radiance field should be integrated. As a result, it can show the same scene at multiple levels of sharp detail. For video synthesis, Pumarola et al. [130] and Li et al. [131] extended the NeRF to dynamic objects with an additional parameter, time t.

The reconstruction and view synthesis processes involve constructing, rendering, and displaying. Obtaining 3D reconstruction through light fields provides researchers with a bright outlook for real-time augmented reality and virtual reality. In addition, light fields provide insights into various view synthesis methods.

### Industrial applications

As mentioned earlier, Lytro and Raytrix have industrialized light field acquisition devices. Other companies, such as FoVi3D [132] and Japan Display [133], have also produced light field projection solutions. Unlike for the Lytro camera, in which a microlens array is placed in front of an image sensor, Wooptix [134] reduced the resolution trade-off by using a liquid lens [135] of the optical chain in front of the sensor to make it possible to change the focal planes quickly, providing full resolution of the sensor in real time. Furthermore, Google published many patents related to light field capturing [136–138]. It also published “Capturing Light Field Images with Uneven and/or Incomplete Angular Sampling” [139] in 2018. It designed a camera to capture light field images with uneven and incomplete angular sampling. The results showed an improvement in not only the spatial resolution but also the quality of the depth data.

For light field display, Avegant [140], Leia [141], Light Field Lab [142], Dimenco [143], and Creal [144] facilitated realistic digital photographs with display screens. Likewise, Looking Glass Factory [145] created a light field image display providing 45 different viewpoints as long as the viewer is within a 58° viewing cone, and it is far less expensive than previous products and, therefore, more attainable. In 2020, Sony published two light field-related technologies: 3D Spatial Reality Display Technology [146] and Atom View [147]. The former tracks the position of the eyes of the user, enabling the user to see real-world images or creations in 3D. In addition, it achieves a relatively high resolution and glasses-free 3D using real-time light field rendering technology. Atom View is an application in volumetric virtual production through point-cloud rendering, editing, and coloring. It can digitize space instead of a physical set and, therefore, can reproduce locations and sets. These light field display solutions are physical products, and light fields can also be applied in immersive online experiences. For instance, OppenFuture [148] provides Hololux Light Field Solution, which focuses on 3D reconstruction. It can reconstruct complex materials at full angle, and it is closely working with e-commerce companies to enhance the shopping experience. Furthermore, Google has produced its glasses-free light field display technology, Project Starline [149], which is used for real-time communication. People can communicate with each other as if they are sitting across from each other. However, Project Starline relies on custom-built hardware and highly specialized equipment.

The above products solve problems in two important parts of the light field imaging pipeline: acquisition and display. In the near future, one can expect more-portable devices for capturing light fields to emerge. In addition, the use of light field displays may extend from fixed screens to display extremely small or extremely large pictures and can further benefit medical microscopy or cinematic displays. Finally, the light field can contribute to closer-to-truth communication, which should make the “smart life” more attainable.

## Conclusions

Depth estimation, which is essential for light field applications, was introduced. Then, the trend of light field applications was evaluated in terms of editing, enhancement, reconstruction, and current industrial products. The light field has been a research focus in computer graphics since 1996 and has progressed into the commercial market since 2010. Starting in 2010, the number of publications has increased rapidly, showing that many researchers are exploring the potential applications of light fields. These studies emphasize the critical role of the light field in enhancing visual experience. However, they require considerable expertise in utilizing light field technology. Therefore, there are still many human-light field interaction challenges to address not only in holography and augmented reality, but also in free image editing and interactive 3D reconstruction. Overall, light field imaging is commercially practical for businesses and individual users.

## Data Availability

All data analysed during this study are included in this published article.

## Abbreviations

1D: One-dimensional
2D: Two-dimensional
3D: Three-dimensional
4D: Four-dimensional
5D: Five-dimensional
AWS: Active wavefront sampling
CNN: Convolutional neural network
6-DOF: Six-degree-of-freedom
EPI: Epipolar plane image
NeRF: Neural radiance field
POBR: Partially occluded boundary region
PSNR: Peak signal-to-noise ratio
RGB: Red, green, and blue
SAI: Sub-aperture images
SPO: Spinning parallelogram operator
SR: Super-resolution
